# Examining the Role of AI in Changing the Role of Nurses in Patient Care: Systematic Review

**DOI:** 10.2196/63335

**Published:** 2025-02-19

**Authors:** Inas Al Khatib, Malick Ndiaye

**Affiliations:** 1 Department of Industrial Engineering College of Engineering American University of Sharjah Sharjah United Arab Emirates

**Keywords:** artificial intelligence, AI, nursing practice, technology, health care, PRISMA

## Abstract

**Background:**

This review investigates the relationship between artificial intelligence (AI) use and the role of nurses in patient care. AI exists in health care for clinical decision support, disease management, patient engagement, and operational improvement and will continue to grow in popularity, especially in the nursing field.

**Objective:**

We aim to examine whether AI integration into nursing practice may have led to a change in the role of nurses in patient care.

**Methods:**

To compile pertinent data on AI and nursing and their relationship, we conducted a thorough systematic review literature analysis using secondary data sources, including academic literature from the Scopus database, industry reports, and government publications. A total of 401 resources were reviewed, and 53 sources were ultimately included in the paper, comprising 50 peer-reviewed journal articles, 1 conference proceeding, and 2 reports. To categorize and find patterns in the data, we used thematic analysis to categorize the systematic literature review findings into 3 primary themes and 9 secondary themes. To demonstrate whether a role change existed or was forecasted to exist, case studies of AI applications and examples were also relied on.

**Results:**

The research shows that all health care practitioners will be impacted by the revolutionary technology known as AI. Nurses should be at the forefront of this technology and be empowered throughout the implementation process of any of its tools that may accelerate innovation, improve decision-making, automate and speed up processes, and save overall costs in nursing practice.

**Conclusions:**

This study adds to the existing body of knowledge about the applications of AI in nursing and its consequences in changing the role of nurses in patient care. To further investigate the connection between AI and the role of nurses in patient care, future studies can use quantitative techniques based on recruiting nurses who have been involved in AI tool deployment—whether from a design aspect or operational use—and gathering empirical data for that purpose.

## Introduction

### Background

The science and engineering field of artificial intelligence (AI) is concerned with the theory and application of creating systems that display the traits we identify with intelligence in human behavior [1]. The years 2000 to 2015 saw an upward trend in the growth of AI. With dramatic revolutions influenced by both ideas and methodologies, the progress of AI has promoted the development of human civilization in our day and age. However, due to its interdisciplinary nature and rapid expansion, AI is a discipline that is challenging to fully comprehend and is getting more and more flexible from the standpoint of reference behavior [2].

The previous decade was defined by AI, and the upcoming one will most likely also be defined by it. Systems that exhibit intelligent behavior by analyzing their surroundings and acting with some autonomy to accomplish predetermined goals are referred to as AI systems. Greater accuracy is needed to have relevant and fruitful discussions on AI because it encompasses so many different methodologies and circumstances. Arguments regarding straightforward “expert systems” that serve advising functions, for instance, must be separated from those about sophisticated data-driven algorithms that make conclusions about specific persons automatically. Similarly, it is crucial to distinguish between arguments regarding hypothetical future advancements that may never materialize and those regarding actual AI that already has an impact on society today including the nursing practice [3].

Numerous ideas, including computing, developing software, and transmitting data, are built on AI. Machine learning (ML), deep learning, natural language processing (NLP), voice recognition, robots, and biometric identification are examples of technologies that use AI. AI is used in a wide range of industries, including the health care, industrial, and automotive sectors as well as corporate organizations. AI also provides several benefits that help it become increasingly popular across numerous industries. AI-powered machines are accurate and efficient, can do many tasks at once, and their work costs less than a human’s. However, there are other issues with AI that make it difficult to use. Technology, security, and data issues are common with AI, and if users do not comprehend the system, mishaps may occur. The expanded use of AI has changed several industries by improving organizational effectiveness and enabling data security [4].

AI in nursing is revolutionizing the field by enhancing patient care, improving efficiency, and reducing the workload on nurses. AI-powered tools and applications enable real-time monitoring of patient’s vital signs, predicting potential health deteriorations, and providing alerts for immediate intervention. AI algorithms can analyze large volumes of patient data to assist in accurate diagnosis and personalized care plans [5]. Moreover, AI chatbots and virtual assistants support administrative tasks, such as scheduling and documentation, allowing nurses to focus more on direct patient care [6]. By automating routine tasks and providing decision support, AI empowers nurses to deliver higher quality care with greater precision and efficiency [7].

### Research Rationale and Aim

The rationale behind this research is to investigate how the increasing use of AI in health care affects the role of nurses in patient care. As AI technologies become more integrated into health care systems, understanding their impact on nursing practice is crucial. AI’s applications, ranging from clinical decision support to operational improvements, promise to transform various aspects of health care, including nursing. By examining whether AI has led to changes in the nursing role or is likely to do so, this research aims to provide insights into how these technologies influence nursing responsibilities and practices. The aim of this review is to explore the evolution of AI as a technology through its various developmental phases. In this systematic literature review, we examine the different applications and deployments of AI in the nursing field. The primary research question addressed is “How will AI transform the role of nurses in patient care?”

### Research Significance

The review offers important perspectives on how AI is transforming the roles and duties of nurses in patient care. This understanding is essential for adapting nursing education, training, and practice to align with evolving technological advancements. By identifying how AI impacts nursing roles, the research can guide the effective implementation of AI tools in health care settings. It highlights the importance of involving nurses in the development and deployment of AI technologies to ensure that these tools enhance rather than disrupt nursing practice. The findings can inform health care policies and training programs by emphasizing the need for ongoing professional development and support for nurses as they integrate AI into their workflows. This ensures that nurses are prepared to leverage AI effectively while maintaining high standards of patient care. The study contributes to the existing body of knowledge on AI in health care and sets the stage for future research. It opens avenues for quantitative studies and empirical data collection to further explore the relationship between AI and nursing roles, providing a foundation for evidence-based practice and decision-making. Overall, this research is important for its potential to enhance the understanding of AI’s impact on nursing practice, guide effective technology integration, and shape the future of nursing education and policy.

## Methods

### Overview

A well-defined review protocol (Textbox 1) was established at the outset of the research to guarantee that the review process is transparent, reproducible, methodical, and provides a clear roadmap for conducting and reporting the review.

**Systematic literature review protocol.**

- Title: confirm a clear, descriptive title for our systematic review.
- Background: explain the rationale behind this research, why it is needed, and its significance. In addition, define the main research aim and research question of the systematic literature review.
- Eligibility criteria: define what was included and excluded from the review inclusive of the search time frame.
- Information sources: list the database searched for academic sources and the gray literature used.
- Search strategy: detail the search terms used and justification of their use.
- Study selection: describe the process for screening that involved reviewers and selecting studies.
- Data extraction and analysis: define the data extraction process and the outcome of data analysis.

This paper presents the findings of a thorough analysis and critical assessment of the pertinent literature using systematic database-searching approaches. The critical assessment in this systematic literature review involves evaluating study quality, relevance, biases, findings synthesis, and implications. This systematic review draws information from credible industry sources as well as published, peer-reviewed English language papers. To comprehend the development of this idea, we consulted trustworthy industry publications known as “gray paper” and the Scopus database covering the last 33 years (ie, 1991-2024). A total of 53 sources comprised of 50 peer-reviewed academic articles, 1 conference proceeding, and 2 reports were included in the research.

**Figure 1:**
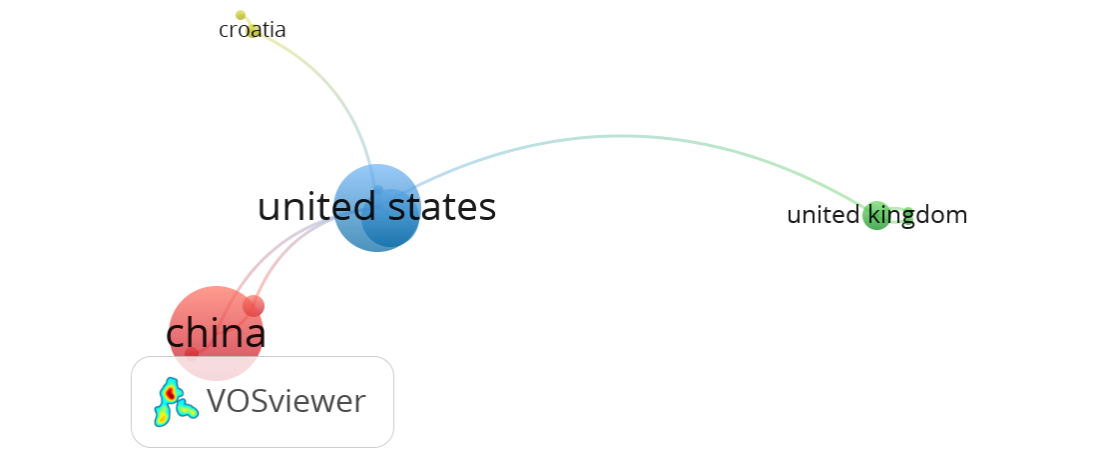
Most countries with research subject “artificial intelligence applications in nursing”—network visualization.

After completing a bibliographic analysis, a suitable keyword search strategy was chosen, such as “AI applications in nursing,” and the search was restricted to the previously specified time frame. On the basis of the outputs of the Scopus database, which have been used in this study to build the various bibliometric maps, bibliometric networks were created using the VOSviewer program (Centre for Science and Technology Studies). After exporting the sophisticated Scopus-based search results to the VOSviewer program, a network visualization was created, as shown in Figure 1, to show how the authors in this area are related to each other through publications on this subject.

In the network visualization illustrated in Figure 1, the United States, followed by China and the United Kingdom, has been publishing journals about AI applications in the field of nursing, demonstrated by the weight of those countries’ representation. Furthermore, the links between those circles indicate that the relatedness of the journals in terms of cocitation links also illustrates that other countries’ journal publications relied on the US publications. A VOSviewer mapping was then done using “AI applications in nursing” as the keyword.

This has prompted the expansion of the search keywords; Table 1 represents a series of search strings focusing on various aspects of AI and its applications, particularly in nursing and related technologies. Alongside, the rationale for each search string is provided along with the number of academic journals found for each. Each search string is designed to capture specific facets of AI to ensure a comprehensive and inclusive exploration of relevant literature. By using these specific search strings, the research ensures a thorough and targeted review of the literature across different aspects and applications of AI, with a special focus on health care and nursing.

**Table 1:** Search string in the Scopus database (N=2870).

Search string	Justification	Results, n (%)
“AI” AND “technology” AND “in” AND “nursing”	This broad search term is used to gather general information and foundational literature on AI^a^, which will provide a broad understanding and context for more specific searches.	164 (5.71)
“Artificial” AND “Intelligence” AND “applications” AND “in” AND “nursing”	This phrase search ensures that both terms are explicitly present, helping to find more specific and relevant documents that discuss AI in a detailed manner.	226 (7.87)
“AI” AND “use” AND “in” AND “nursing”	This search string targets the literature that explores the application and impact of AI technologies specifically within the field of nursing, ensuring relevance to health care.	162 (5.64)
“Nursing” AND “AI” AND “applications”	By including these terms, the search focuses on technological advancements and their practical uses in the nursing profession, broadening the scope beyond just AI.	105 (3.65)
“Evolution” AND “of” AND “Artificial” AND “Intelligence” AND “Approaches” AND “in” AND “nursing”	This search string aims to find the literature on the historical development and various methodologies within AI, providing context and background on how AI approaches have changed over time.	6 (0.20)
“Symbolic” AND “AI” AND “Approach”	Symbolic AI is a specific paradigm within AI research. This search will help identify works focused on this particular approach, which is crucial for understanding different AI methodologies.	620 (21.60)
“Data-Driven” AND “AI” AND “Approach” AND “in” AND “nursing”	Data-driven AI approaches, including ML^b^ and neural networks, are fundamental to modern AI. This search focuses on the literature that discusses these data-centric methodologies.	2 (0.06)
“Artificial” AND “General” AND “Intelligence” AND “Approach” AND “in” AND “nursing”	AGI^c^ represents a more advanced and comprehensive form of AI. This search will help identify research on AGI, exploring its potential and challenges.	10 (0.34)
“Artificial” AND “Intelligence” AND “application” AND “in” AND “nursing”	This search string is designed to find specific case studies and examples of how AI is being applied in nursing, providing practical insights and real-world applications.	230 (8.01)
“Rothman” AND “Index” AND “Use” AND “for” AND “Patient” AND “Acuity” AND “and” AND “Risk”	The Rothman Index is a specific tool used in health care. This search targets the literature on its use and effectiveness in assessing patient acuity and risk, relevant for AI applications in patient monitoring.	1 (0.03)
“Social” AND “robots” AND “use” AND “in” AND “nursing”	Social or companion robots are an emerging area within AI and robotics. This search aims to find the literature on their use, particularly in providing care and support in health care settings.	104 (3.62)
“TeleRobots”	Telerobots are used for remote operations, which can be highly relevant in health care for tasks such as remote surgery or patient care. This search focuses on this specific technology.	105 (3.65)
“Natural” AND “Language” AND “Processing” AND “in” AND “nursing”	NLP^d^ is a key area within AI, crucial for developing systems that can understand and process the human language. This search targets the literature on NLP, which has substantial applications in health care communication and data analysis.	256 (8.91)
“Robotic” AND “Process” AND “Automation” AND “in” AND “nursing”	RPA^e^ is a form of business process automation technology based on AI. This search string is aimed at finding the literature on how RPA can be applied in health care operations and administration.	13 (0.45)
“Machine” AND “Learning” AND “use” AND “in” AND “nursing”	ML is a core component of AI. This search aims to gather comprehensive literature on ML techniques and their applications across various domains, including health care.	208 (7.24)
“nurse” AND “role” AND “transformation”	AI is driving significant changes in health care by automating tasks, supporting decision-making, and transforming traditional nursing functions. The focus on “role” and “transformation” highlights how nurses’ responsibilities are evolving due to AI integration, requiring new skills and altering patient care practices. These keywords enable a targeted exploration of the evolving landscape of nursing in the context of AI-driven health care.	658 (22.92)

^a^AI: artificial intelligence.

^b^ML: machine learning.

^c^AGI: artificial general intelligence.

^d^NLP: natural language processing.

^e^RPA: robotic process automation.

### Data Collection and Analysis

#### Academic Search

The inclusion and exclusion criteria are presented in Textbox 2.

**Inclusion and exclusion criteria.**

**Inclusion criteria**

- Empirical studies, conference proceedings, and reports
- Papers with clear research questions and objectives on the application of artificial intelligence in the field of nursing
- Time period: from 1991 to 2024 (ie, 33 years)
- Papers published in the English language

**Exclusion criteria**

- Conceptual papers, editorials, academic book sections, and literature reviews
- Industrial sectors other than health care
- Publications before 1991
- Other languages

### Data Selection

#### Overview

Information from all the 401 references was compiled in a soft copy folder. These references were independently reviewed by the main author, who selected the final list of papers to be analyzed. The first author examined the articles’ topics and content and used our criteria for inclusion and exclusion of material to eliminate papers whose research questions were not fully aligned with the scope of this review. The second author upheld the main author’s decision to exclude the resource from the study. The inclusion criteria are the characteristics that must exist to be included in this study, while the exclusion criteria are those characteristics that disqualify a data source from inclusion in the paper, which leads to the identification of 50 relevant journals published between 1991 and 2024.

#### Gray Literature Search

With regard to searching supplementary sources, it was imperative to expand the search radius to include official newspapers and reliable industry sources because the topic of this study is a prominent issue in industry trends. These sources capture the expert opinions of subject matter experts and produce additional information from trustworthy sources such as the European Parliamentary Research Service and National Bureau of Economic Research. Pertinent supplemental sources were found as a result, and the study report examined them all.

The critical assessment of the included studies was conducted through a self-rating process by 2 authors. Each author independently reviewed and appraised the quality of the studies based on predefined criteria relevant to the study designs, including risk of bias, methodology, and relevance to the research question. The 2 authors then compared their ratings, and any discrepancies were discussed and resolved through a consensus. This self-rating approach was used to streamline the evaluation process while ensuring consistency in the appraisal.

Furthermore, Table 2 provides a summary of the selected data in the systematic review literature by type.

**Table 2:** Systematic review analysis summary by type.

Reference type	Values, n (%)
Journal article	50 (94)
Conference proceeding	1 (2)
Report	2 (7)

#### Thematic Analysis

**Figure 2:**
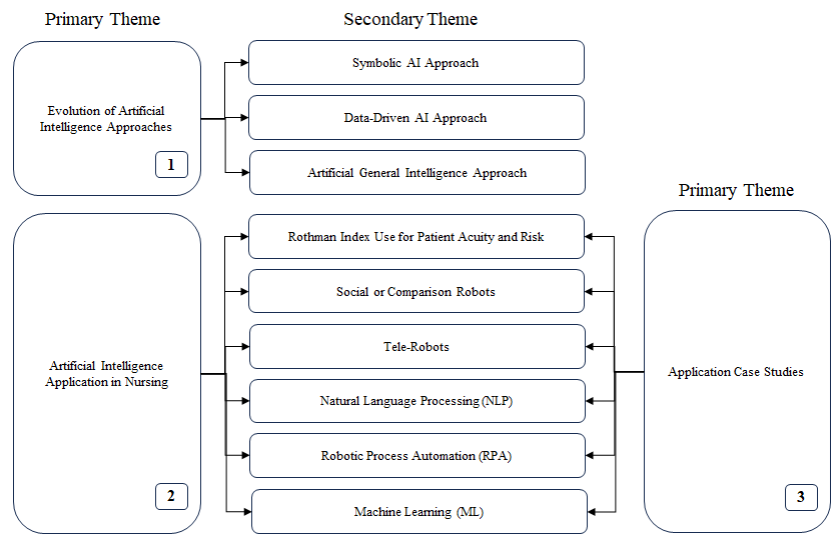
Primary and secondary themes in the systematic literature review. AI: artificial intelligence.

The study made use of secondary data sources; these resources offer details on the numerous ways AI is being used in nursing and how this is changing the role of nurses. The collected material was examined using a thematic analysis approach. The information gathered from the literature study was carefully reviewed by 2 independent reviewers and categorized in accordance with the primary themes and supporting themes that surfaced. Common patterns, trends, and important discoveries must be found to comprehend the connection between the findings. The primary themes that were identified from the literature are mapped in Figure 2.

This methodology was consistently applied throughout the paper and the results produced will be discussed in the subsequent section.

A thematic analysis was conducted explicitly designed to assess the impact of AI on the role of nurses. The use of the keyword “nurse role transformation” triggered an extensive review of the relevant literature, where we systematically screened for statements or data points related to the interaction between nurses and AI-based technologies. For example, we examined whether articles explicitly discussed shifts in task allocation, automation of routine functions, or changes in decision-making responsibilities. Furthermore, we used a matrix systematization process where each identified AI technology was mapped against the roles and responsibilities traditionally held by nurses, as well as any newly emerging roles due to the technology’s integration. This allowed us to systematically capture how AI is transforming the scope of nursing practice, such as by enabling nurses to focus more on patient-centered care while AI systems manage data analysis or administrative tasks.

In the Results section, we expanded on this by introducing a subtheme explicitly titled “roles of nurses and role transformation.” This subtheme synthesized case studies and literature findings that demonstrated specific examples of role shifts, such as how AI-assisted diagnostic tools are enabling nurses to participate more actively in clinical decision-making or how AI-driven administrative systems reduce the clerical burden on nurses. These shifts were categorized into functional changes (such as delegation of monitoring tasks to AI systems) and strategic changes (such as enhanced involvement in decision-making processes due to AI’s real-time data processing capabilities).

This systematic approach clarifies the origin of our conclusions, making it explicit that role transformation insights were derived from both the literature and our thematic analysis, supported by the matrix framework developed during the study.

## Results

### Results of Data Collection

**Figure 3:**
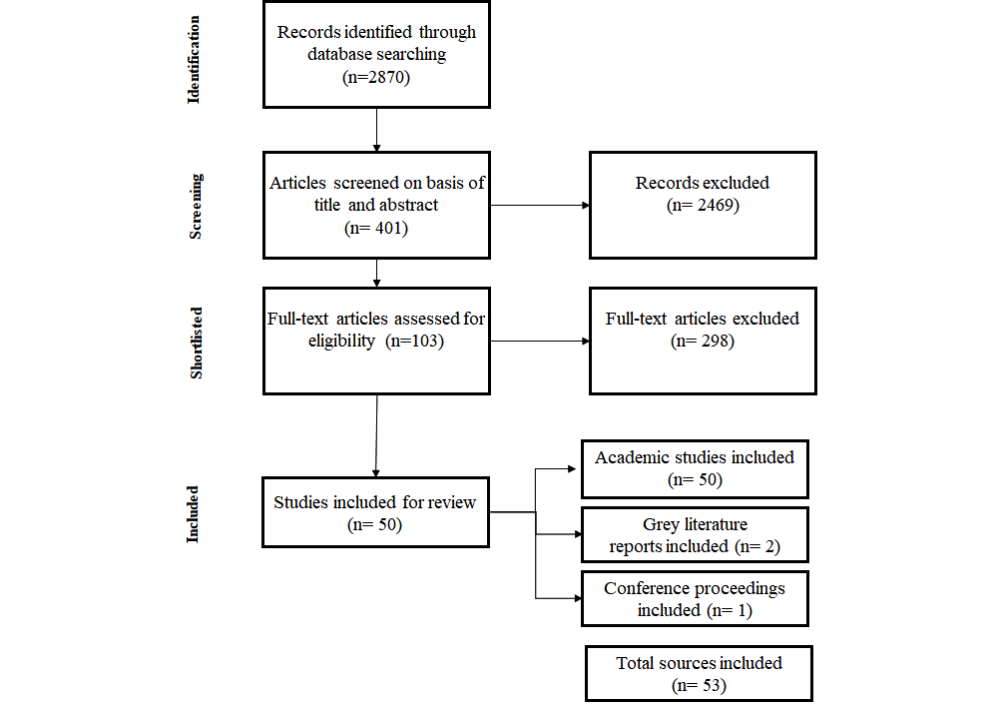
The systematic article selection process for this review.

The outcome of the journal searches yielded 2870 results, out of which 401 sources were shortlisted to be analyzed further, as demonstrated in Figure 3 and elaborated further in the subsequent section.

### Evolution of AI Approaches

#### Wave 1: Symbolic AI Approach

Expert systems and symbolic AI are 2 terms used to describe the initial wave of early AI approaches. In this case, human specialists develop exact rule-based processes, or “algorithms,” that a computer may use to decide how to respond intelligently to a particular circumstance. A variation of this strategy called fuzzy logic allows for varying degrees of confidence in a scenario, which is helpful for capturing intuitive knowledge and enabling the algorithm to make wise judgments in the presence of numerous, uncertain, and interconnected factors. In contexts with rigorous rules and variables that are clear-cut and measurable, which do not vary significantly over time, symbolic AI performs well. These techniques may seem old, yet they are still used today [3].

#### Wave 2: Data-Driven AI Approach

The second wave of AI consists of more modern, “data-driven” methodologies that have advanced quickly over the past 2 decades and are primarily to blame for the present rebirth of AI. These do away with the first wave AI’s reliance on human specialists by automating the learning of algorithms. Artificial neural networks (ANNs) are modeled after how the brain functions. The translation of inputs into signals that are then sent across a network of synthetic neurons to produce outputs that are seen as reactions to the inputs. ANNs can handle increasingly complicated issues by adding additional neurons and layers. An ANN with several layers is simply referred to as deep learning. ML is the process of changing a network so that its outputs are seen as useful or intelligent answers to its inputs. By using evolutionary concepts to produce slow improvements in huge populations of ANNs or by making gradual changes to individual ANNs, ML algorithms may automate this learning process [3].

#### Wave 3: Artificial General Intelligence Approach

The third wave of AI is a hypothetical term for potential future waves of AI. First and second wave approaches are referred to as “weak” or “narrow” AI in that they can act intelligently in just certain contexts and issue domains, whereas “strong” or “general” AI refers to algorithms that can act intelligently across a variety of contexts and problem domains. With existing technology, such artificial general intelligence is not feasible and would need paradigm-shifting development. Advanced evolutionary techniques, quantum computing, and brain emulation are a few possible strategies that have been considered. Although self-explanatory and contextual AI may have modest goals compared to other futuristic AI types, their potential influence and implementation challenges should not be understated [3].

### AI Applications in Nursing

There is still much to learn about the innovative and intricate challenges surrounding AI. For health care businesses to best serve patients and physicians, AI must be fully used [8]. In subsequent sections, we have discussed examples of AI applications in nursing.

#### Rothman Index Use for Patient Acuity and Risk

The level of acuity and risk of a patient are both reflected by the Rothman Index. The electronic medical record (EMR) data connected to 26 variables, including 11 graphically represented nurse evaluation measures, are used to determine scores. The introduction of the Rothman Index was accompanied by doubts regarding its accuracy and dependability in delivering results that could be put into practice. At first, there was not enough peer-reviewed research on the technology to persuade nurses and other professionals that the outcomes would improve patient care. The capacity of nurses to affect patient care is crucial, as evidenced by a recent study indicating that the Rothman Index’s performance is positively impacted by nurses’ evaluation data [9].

#### Social or Companion Robots

Social robots are made to react to human interactions in a way that makes them human. Sophia (Hanson Robotics) is an illustration of a social robot designed as a companion for older adults that shows the possibility of technological developments to enhance how robots operate. Robots are being developed by researchers all across the world to enhance therapeutic telemedicine applications, reduce suicide rates, and more. The role of nurses in providing care will evolve as robots learn to carry out nursing tasks such as ambulation support, vital sign assessment, drug administration, and infectious disease procedures. According to research, nonnursing chores and activities take up between 8% and 16% of nursing time. With robot assistance, nurses will be able to reclaim this time and devote it to patients more. Does this imply that nursing is doomed to extinction? Absolutely not; in fact, the exact reverse is happening. Robots created and used for patient care and older adults’ assistance are being developed by nurses. Nurses can receive assistance from the robots at the bedside or in the community [9].

#### Telerobots

Telerobots can support health care professionals who are at “high risk for infection due to routine patient contact, handling of contaminated materials, and challenges associated with safely removing protective gear.” Furthermore, telepresence robots support nurse-led treatments for the promotion of healthy lifestyles and the management of chronic illnesses by combining an initial in-home visit to launch the health care program with subsequent remote telehealth visits made at the patient’s home. Data are gathered on participant health outcomes as well as the robot intervention’s usefulness and pleasure [9].

#### NLP Approaches

A language is a system of rules or a collection of symbols that are integrated and used to express ideas or disseminate information. NLP serves users who lack the time to learn new languages or become proficient in their current ones because not all users have a strong background in machine-specific language. In actuality, NLP is a branch of linguistics and AI whose goal is to enable computers to comprehend assertions and words spoken in human languages. It was developed to make the user’s job easier and to fulfill their desire to speak to a computer in natural language. It can be divided into 2 categories: natural language generation and natural language understanding, which progresses the task of understanding and producing the text [10]. In nursing, NLP assists in nursing practice and decision-making. Using NLP, it is possible to analyze nursing records, spot patterns and trends in patient care, and gain knowledge that will enable nurses to give patients more individualized and effective treatment [11].

#### Robotic Process Automation

Robotic process automation (RPA) is a method that uses robotics as a set of techniques for the operation and use of automata (ie, robots) in the execution of multiple tasks in place of humans as the standard, method, or system. RPA results in the automatic execution of administrative, scientific, or industrial tasks. RPA tools are a set of methods intended to enhance productivity by automating and minimizing the number of repetitive jobs. The inclusion of AI algorithms and techniques to the use of RPA enhances the accuracy of the execution of automated procedures [12].

#### ML Algorithms

The study of algorithms and statistical models that computer systems use to carry out a particular task without being explicitly taught is known as ML. There are several daily-use programs that incorporate learning algorithms. One of the reasons a web-based search engine like Google works so well every time it is used to search the internet is because of a learning algorithm that has mastered the art of ranking websites. These algorithms are used for several different tasks, including data mining, image processing, and predictive analytics. The major benefit of ML is that once an algorithm understands how to use data, it can carry out its task autonomously [13].

The study of ML considers how to automatically generate reliable predictions from complicated data. It is strongly tied to contemporary statistics, and in fact, statisticians have contributed many of the most brilliant concepts in ML (eg, the lasso, trees, and forests). However, the ML community has been more focused on the single objective of maximizing predictive performance, in contrast to statisticians who have frequently concentrated on model inference—that is, knowing the parameters of their models (eg, testing on individual coefficients in a regression). “Out-of-sample” tests, which assess how well a model trained on 1 dataset would predict fresh data and serve as the benchmark for the whole ML discipline [14].

### Application Case Studies

#### Overview

Relevant case studies and examples from the literature were used to highlight the involvement of nurses in the identified AI applications or tool deployments and to assess whether there is a change or anticipated elimination to the role of nurses in patient care. These case studies focused on certain types of AI applications that are tied to certain activities carried out by nurses as part of their core patient care role. The following examples were selected due to their applicability, importance, and contribution to the comprehension of the study’s subject.

#### Rothman Index Use

The Specialized Workforce for Acute Transport (SWAT) team of nurses trained in critical care, advanced cardiovascular life support, and trauma care at Yale New Haven Hospital is a real-world example of using the Rothman Index technique. When signs point to a patient’s condition deteriorating, they immediately receive alerts on their mobile phones. The SWAT team looks through the EMR, evaluates the patient as needed, and works together with clinical nurses and other medical personnel on pertinent areas of treatment. SWAT nurses identify as “a second pair of eyes” in their own description. The index’s information came from widely available nursing literature. Given that the index is updated in real time from the EMR, timely submission of nurse assessment data is essential for the computation and value of the index score [9].

#### Social or Companion Robots

Sophia is an illustration of a social robot designed as a companion for older adults that shows the possibility of technological developments to enhance how robots operate. Sophia had a refurbishment in 2018 that included movement features, and she is currently the first robot to be granted citizenship in a nation (ie, Saudi Arabia) [9]. The next generation of social robots with cutting-edge AI is the LOVOT robot. The social robot LOVOT was well received by most patients with dementia. LOVOT exhibited beneficial impacts, improved communication, and promoted social engagement. Although LOVOT had no appreciable benefits on social well-being, it provided individuals with a break from daily living. Following their interactions with LOVOT, some residents experienced emotional overstimulation. The social robot was embraced by medical specialists and nurses, who saw LOVOT as a new tool for working with patients with dementia as a supporting tool and not as a replacement of the care provider’s role [15].

A major factor in concentrating on the older adults is AI. It can, for instance, strengthen the bonds between older adults and their relatives or care teams. Furthermore, an AI chatbot can converse with the older adult without any difficulties and may remind them of important dates, such as medication intake and medical exams. Many of the AI smartphone apps available now have the ability to screen wellness data in a less intrusive manner, including daily activity, food, and, shockingly, older adults lifestyle choices. In certain situations, it could be helpful to anticipate and, thus, prevent any potential hypertension or irregular heart rate. In essence, robotic “pets” are also helping to improve patient attention while also assisting in the battle against emotions of loneliness. One such model, called Tombot, is a small, dog-like device designed to relieve anxiety in patients with dementia. Its head movements, appearance, and swinging tail are remarkably similar to those of the genuine dog, giving owners the impression that they have their own pet to truly concentrate on. The care of the older adults is one of the problems that low-income nations are experiencing. The global shift of older populations has worsened the shortage of trained people in the older adults health care context. Given that the number of older people worldwide is predicted to almost treble in the next 3 decades, there may be a greater need for older adult care [16].

#### Telepresence Robots

Health care professionals who are at “high risk for infections due to routine patient contact, handling of contaminated materials, and challenges associated with safely removing protective gear” are the focus of Tele-Robotic Intelligent Nursing Assistant, a remote-controlled robot, at Duke University Pratt School of Engineering and School of Nursing. Noting that Tele-Robotic Intelligent Nursing Assistant is 20 times slower than a nurse, it presently completes around 60% of the preset nursing tasks in the nursing simulation laboratory where it is being evaluated. Results from individuals getting telehealth coaching from home reveal that patients and clinicians alike find satisfaction in the mix of live face-to-face interventions and robotic telehealth visits. Designing meaningful treatments that can take use of new technology requires the nurse to have a key part in the development and execution of telehealth robots [9].

Inpatient rooms at the nonprofit, tertiary, 958-bed Cedars-Sinai Hospital in Los Angeles, California, are equipped with Alexa robots created by Amazon to serve as virtual nurse aides. To support patients with their daily routines, Alexa fulfills the monotonous activities performed by nurses. She also assists in answering medical queries and reminds patients to take their pills on time [17].

#### NLP Use

The most commonly used AI functions in studies of AI-related nursing activities were profiling and prediction, followed by assessment and evaluation. Virtual reality teaching interventions and learning successes were beneficial to nurses because they provided a safe learning environment with the possibility of multiple tries, overcoming challenges, the ability to consolidate knowledge, and professional efficacy [18]. Furthermore, the use of chatbots improves student learning compared to traditional teaching techniques [19], acting as supporting tools to nursing educators rather than eliminating the entirety of their role.

NLP is used by triage nurses to register and categorize patients based on their speech. When conducting triage activities, RMIS-AI is quicker than using the manual input approach, which decreases the time it takes to register patients and classify them. To address the existing level of subpar sensitivity and accuracy provided by nurses, technological augmentation is necessary [20].

Primary care nurses are faced with increasing demands from patients who have wounds from a variety of sources. Both nurses and patients can benefit from a chatbot that provides information properly verified on the basis of evidence. By providing instructions on the suggested wound dressing techniques for each type of wound, BOTCURATIVO, a chatbot, seeks to assist nonspecialists in the management of wounds. A reasonable degree of content validity was attained by the script that was created and implemented into the chatbot prototype. The chatbot’s usability was seen as being good, which increased the device’s credibility. Noting that regardless of their specialty, the nurse will always undertake wound management tasks [21].

Most people lack the medical knowledge necessary to investigate or understand the severity of their conditions or symptoms. In this regard, NLP is essential to health care. These chatbots gather health information from patients, analyze it, and recommend actions to patients based on more pertinent knowledge of their physical conditions. Health care chatbots similar to NOVA—a virtual nursing assistant driven by AI—are helpful in the medical field because they help patients and point them in the direction of the right resources. When consumers or patients look up answers to inquiries they have about their health on the internet, chatbots are more helpful. A user of this program may text requests for health care and may receive pertinent health advice in return. A chatbot can provide medical information, including illness symptoms and treatment options. Patients receive professional guidance in real time, and their personal and medical data are kept in a database for future research. The number of AI-powered health care apps has significantly increased recently. Consequently, there are shorter wait times in offices, which saves money and energy. Patients may be helping in their own place and at their own speed while learning medical knowledge. User input is received by the system via text or speech data. The system interprets the input data. The virtual nursing help system may be accessed by the user who can also send an inquiry to it. The output that the system produces is a list of user symptoms and suggested diagnoses. In the area of virtual nursing help, the suggested system serves as the user’s personal assistant. The created bots are useful for keeping track of patient information. The technology can also help numerous people at once [22].

In MobiGuide, the role of nurses in creating, providing, and assessing eHealth-based services was examined with an emphasis on atrial fibrillation home monitoring. To obtain suggestions, warnings, and reminders about drugs and measures that they should conduct, patients were given smartphones and electrocardiogram sensors. This mobile decision support system was regularly updated by a backend system. Health care professionals are supplied patient data so they may view it and take appropriate action. With their participation in the design of the caregiver interface, responsibility for the enrollment phase (ie, including patient training), daily data checks, triage of patient concerns, and patient interviews about their experiences with the system, nurses play a key role in such settings [23].

The Smart Wearable Physiological Signal Measurement Integration System is used in home care, nursing homes, and other health care settings to continuously monitor their patients’ vital signs, which enables nurses to see early warning indicators of deterioration and take quick action to stop unfavorable outcomes. When a patient exits a specified area or when vital signs suggest an emergency, the system may notify the care providers, improving patient safety in nursing homes and home care settings [24].

The development of indoor positioning technology has made it feasible to track the movement of mobile medical equipment within a hospital ward, including patient monitors and electrocardiography devices. Nurses can quickly detect and locate a gadget with the help of an item tracking system, particularly while they are getting ready for a medical procedure or shift change. Given that nurses typically have a heavy workload, it would be ideal to give them access to a well-liked mobile app with an intuitive search interface that they can use on a regular basis. To help with this, DBOS, a dialogue-based object query system, offers voice and text inquiry services to nurses while mimicking a genuine discussion with users through the chatbot interface of the Line messaging app [25].

The accurate assessment of pain in the neonatal intensive care units is essential due to the high prevalence of painful experiences. Video-based assessment of neonatal pain could be reliably used, as confirmed by the high intrarater and interrater reliability between direct observation and the video-based assessment, as well as the AI method-based performance evaluation, even with various disturbances in real-world neonatal intensive care units. Video-based assessment is viable for neonatal pain assessment in a clinical setting, and the extent of neonatal pain can be evaluated remotely in real time, which can better identify and treat it and thus improve the neonatal pain condition. Video-Based Neonatal Pain Assessment can reduce the stressful surroundings of a clinical setting, the contextual noise, and other elements that could shift the focus of the trainees from the rating. There has been an increasing interest in using ML methods for understanding human behavioral responses to pain based on the analysis of facial expressions, crying sounds, and body movement. Several automated methods have been introduced to automatically assess infants’ pain based on behavioral or physiological pain indicators analysis. Using AI-based neonatal pain assessment, the nursing staff can also use these recordings to judge the pain level by observing the painful procedure video in the nurse station and taking timely intervention measures, which could greatly reduce the bedside observation time and improve work efficiency [26].

#### Robotic Process Automation

Patients with diabetes mellitus face a 15% to 25% lifetime risk of developing diabetic foot ulcers (DFUs). Monitoring and assessing DFUs for complications and healing progress is essential, and this was traditionally performed using manual measurements. A past study compared conventional measurement methods with an AI-powered mobile application for wound imaging, the CARES4WOUNDS (C4W) system [27]. The length and breadth of the wound were the major characteristics measured. C4W measures had good intra- and interrater reliability compared to standard wound measuring. The C4W was a helpful tool for keeping track of DFU wound healing, yet it did not eliminate the role of wound care nurses.

#### Machine Learning

In 1 in 8 to 10 cases where primary care physicians and nurse practitioners used AI, they made better diagnoses, suggesting the potential for raising the standard of dermatologic treatment. The diagnoses showed improvements of 10% and 12% for primary care physicians and nurse practitioners, respectively, indicating a significant positive impact [28].

For long-term patient care, Vitalerter has developed a program that combines advanced biosensors and deep learning to provide contactless and continuous vital sign monitoring, as well as cloud-based early warning protection services. Some of the standout features of these systems are accurate body movement analysis, continuous heart and respiratory rate monitoring, and contactless detection of patients moving out of bed. In the event of an adverse event, the system will automatically sound an alarm to remind nurses to take immediate action and lower the risk of falls, pressure sores, and septicemia [29].

Converting pediatric nursing diagnoses into a digital format and adding them to a case base to evaluate how well the prototype handled these cases allowed for case comparison, retrieval, adaptation, and indexing. Therefore, this study offers a computational tool for the health sector that makes use of case-based reasoning, an AI method. While case-based reasoning is merely another paradigm for problem solving, what sets it apart from other AI approaches is how it differs from them. Rather than relying just on a general understanding of the issue or creating connections between problem descriptions and conclusions, this paradigm can use specific information from past experiences or real problem situations. It is acknowledged that using nursing care systematization necessitates that nurses develop a variety of abilities and adhere to theoretical support to enhance decision-making. Decisions should then be discussed with the patient whenever feasible. The application of these records or technology in various clinical health situations, in which observations about the care needs of patients accompany the decision-making process about the care provided, assists in the subsequent evaluation of the outcomes that are obtained with professional intervention. In this way, it is known that the nursing care systematization collaborates to provide safe, logical, and effective nursing care. Organizing the administration of nursing care and assisting nurses in making decisions is also predicated on ensuring patient safety at various care levels [30].

Table 3 summarizes the thematic analysis and links the respective case studies outlined in this research paper.

On the basis of the themes outlined from the literature review, 81% (13/16) of the AI applications within the nursing fields are in the proof-of-concept phase, with 19% (3/16) of those deployed demonstrating a positive impact on the nursing role within the patient’s journey with the United States leading the way in such research and developments. Furthermore, applications that would enhance or streamline the nurses’ role seem to be focused on the treatment stage, followed by 25% on posttreatment care (ie, recovery). Noting that the applications cover various aspects of the nursing activities from diagnosis, treatment, wound management, education, and training to triaging.

Multimedia Appendix 1 [1-53] provides a clear summary of our systematic review by analyzing 37 sources in terms of key findings, methodology, sample size, potential biases, and validity. This is to ensure the robustness and reliability of the conclusions drawn from the systematic review.

**Table 3:** Outcome of the thematic analysis.

AI^a^ application in nursing and country		Name	Nursing involvement	Status
**Rothman Index**				
	United States (Yale New Haven Hospital)	Rothman Index	Treatment	Operationally deployed
**Social or companion robots**				
	Saudi Arabia	LOVOT	Posttreatment care (ie, recovery)	Operationally deployed
	United States	Tombot	Posttreatment care (ie, recovery)	POC^b^
**Telepresence robots**				
	United States (Duke University Pratt School of Engineering and School of Nursing)	TRINA^c^	Treatment	POC
	United States (Cedars-Sinai Hospital)	Alexa	Treatment	Operationally deployed
**NLP^d^**				
	United States	RMIS-AI	Triage	POC
	United States	BOTCURATIVO	Nurse education and training	POC
	United States	NOVA-a virtual nursing assistant	Diagnosis	POC
	United States	MobiGuide	Posttreatment care (ie, recovery)	POC
	United States	Smart Wearable Physiological Signal Measurement Integration System	Posttreatment care (ie, recovery)	POC
	United States	DBOS^e^, a dialogue-based object query system	Treatment	POC
	United States	VB-AI^f^ NPA	Treatment	POC
**RPA^g^**				
	Singapore	CARES4WOUNDS system, Tetsuyu	Wound management	POC
**Machine learning**				
	United States	Artificial intelligence aid	Diagnosis	POC
	United States	Vitalerter vital sign monitoring	Treatment	POC
	United States	CBR^h^	Treatment	POC

^a^AI: artificial intelligence.

^b^POC: proof of concept.

^c^TRINA: Tele-Robotic Intelligent Nursing Assistant.

^d^NLP: natural language processing.

^e^DBOS: dialogue-based object query system.

^f^VB-AI: video-based artificial intelligence.

^g^RPA: robotic process automation.

^h^CBR: case-based reasoning.

### Roles of Nurses and Role Transformation

The integration of AI technologies into health care has significantly transformed the roles of nurses, shifting their focus from routine tasks to more advanced and patient-centered care [6]. AI systems automate many traditional nursing responsibilities, such as monitoring patient vitals, data entry, and medication management, allowing nurses to prioritize clinical decision-making, patient education, and emotional support. This role transformation not only enhances the efficiency of health care delivery but also enables nurses to engage more deeply in patient care by using AI as a collaborative tool [31]. AI-driven systems support clinical decision-making, triaging, and diagnostic processes, leading to improved patient outcomes and job satisfaction among nurses [32]. Table 4 provides an overview of how AI can transform nursing roles across various functions.

**Table 4:** Traditional nurse role versus artificial intelligence (AI)–driven role transformation.

Traditional nurse role	AI-driven role transformation	Example of AI technology involved	Impact on patient care	Academic reference
Monitoring patient vital signs	AI takes over continuous monitoring, alerting nurses only when intervention is needed	AI-based monitoring systems (eg, wearable sensors and IoT^a^ devices)	Frees up nurses’ time for more personalized, hands-on patient care and reduces error risk through automation	Ross et al [37]
Data entry and record keeping	AI automates data entry, streamlining the EHR^b^ updating process	AI-enabled EHR systems with NLP^c^	Reduces administrative burden, allowing nurses to focus on direct patient care	Zou and Schiebinger [33]
Routine diagnostic procedures	Nurses assist in AI-driven diagnostics, focusing more on patient interaction and explaining results	AI diagnostic tools (eg, image analysis for radiology and pathology)	Enhances the role of nurses as educators, helping patients understand diagnoses and treatments	Ng et al [38]
Medication administration	AI systems manage medication scheduling, and dosing, with nurses overseeing AI-generated plans	Automated dispensing systems and AI-driven dose calculators	Reduces medication errors and ensures timely administration, allowing nurses to focus on patient observation	Shang [39]
Patient triage and assessment	AI aids in triaging by prioritizing patients based on real-time data, allowing nurses to focus on high-priority cases	AI-powered triage systems (eg, in emergency departments)	Increases efficiency in patient care and enhances the accuracy of triage decisions	Govindaraj et al [40]
Clinical decision support	Nurses collaborate with AI systems that provide real-time decision support based on predictive analytics and historical data	AI-based decision support systems (eg, IBM Watson and AI in ICU^d^ for risk prediction)	Empowers nurses to contribute more significantly to clinical decision-making and patient care planning	El-Kareh and Sittig [41]
Health education and counseling	AI tools provide nurses with real-time personalized health data to tailor patient education more effectively	AI-driven patient education platforms (eg, AI chatbots and personalized health apps)	Enhances the nurse’s ability to deliver personalized health education and counseling based on real-time insights	Li et al [42]
Supervision of junior staff	Nurses oversee AI-driven workflows and ensure that AI-generated protocols are followed, focusing more on clinical mentorship	AI systems for task delegation and workflow automation	Enhances leadership roles, allowing nurses to take on a supervisory role and focus on mentorship and training	Rony et al [43]
Wound care and management	AI tools help nurses monitor wound healing through image analysis and predictive algorithms	AI-based wound care imaging systems (eg, predictive models for healing times)	Improves the accuracy of wound assessment, reduces manual checks, and improves patient outcomes	Rippon et al [44]
Patient discharge planning	AI assists in generating discharge plans, predicting postdischarge risks, and automating referrals to follow-up care systems	AI-driven discharge planning tools	Optimizes discharge planning and postdischarge care, reducing the likelihood of readmissions	Jack et al [45]
Emotional support and communication	AI systems can handle administrative tasks, enabling nurses to spend more time on patient emotional support and communication	AI-powered administrative assistants (eg, scheduling systems, automated communication)	Allows nurses to prioritize emotional support and patient communication over routine tasks	Robert [9]

^a^IoT: Internet of Things.

^b^EHR: electronic health record.

^c^NLP: natural language processing.

^d^ICU: intensive care unit.

Recent studies support these findings, showing that AI systems can help optimize workflows, reduce administrative burdens, and allow nurses to contribute more meaningfully to clinical care. Health care AI tools, such as predictive analytics and automated documentation systems, have been shown to improve patient outcomes while minimizing the risk of human error in routine tasks [33,34]. Moreover, AI-based decision support tools in critical care environments enable nurses to make informed decisions quickly, positively impacting patient care quality [35]. These advancements are particularly evident in the transformation of nursing roles, as evidenced in a thematic analysis of health care AI implementations [36].

### Critical Assessment of the Literature

The literature collectively covers a broad spectrum of AI applications, ranging from technical reviews and policy implications to specific domains such as health care and nursing. The mix of older foundational papers and recent studies provides both historical context and insights into current advancements. Practical and policy-oriented papers enhance the literature by addressing real-world applications and implications of AI. However, some biases were identified, particularly in policy reports like the one by Boucher [3], which reflect institutional viewpoints. The focus on health care and nursing in several papers could skew the overall perspective toward these fields. In addition, journals with lower impact factors might have less rigorous peer review processes, potentially affecting research quality.

The synthesis of findings indicates a strong direction toward integrating AI in various fields, particularly health care and nursing. There is a clear emphasis on the transformative potential of AI, along with discussions on challenges and ethical considerations. Comparative studies and reviews highlight the advantages and limitations of different AI approaches, suggesting the need for context-specific solutions. The quality and diversity of the studies imply that AI is a rapidly evolving field with significant interdisciplinary impacts. Practical guides and policy reports emphasize the need for continuous education and ethical considerations in AI deployment. The focus on health care underscores AI’s potential to improve patient outcomes, though it also highlights the importance of rigorous evaluation and context-specific applications.

In conclusion to this section, the systematic literature review provides a comprehensive overview of AI, balancing theoretical foundations, recent advancements, practical applications, and policy implications. While some sources may carry biases or lack depth in certain areas, the collective insights offer valuable guidance for understanding AI’s multifaceted impact as outlined in Multimedia Appendix 2 [1-53].

### AI in Nursing From a Theory and Management Perspective

Numerous theoretical and managerial contexts have debated the use of AI in health systems in general and in tasks associated with the nurses’ role in particular. As an overarching theoretical viewpoint, nurses will continue to provide direct patient care due to nuances in human behavior. The ability to incorporate new tools and technology will be required of nurses. As technology is being incorporated into nursing programs’ curricula, the nursing profession is evolving. Thus, from a management viewpoint, nurses will continue to integrate the data produced by AI tools. They will need to have the skills to incorporate AI findings into evidence-based practice while combining that knowledge with nursing expertise [9].

Despite limitations in identifying numerous pieces of the literature that address the impact of deploying AI, given that many tools and techniques are in project or testing stages, our research contributes to current discussions on contextualized research.

Through this systematic literature review, we attempted to establish a foundation to identify the existing studies from the context emic perspective. By encouraging the research community to focus on “optimal allocation of effort between exploitation and exploration,” looking at theoretical contributions from the periphery will progress management and organization science [46]. We encourage academics to perform empirical studies for the benefit of advancing literature in this arena. From a practical perspective, physicians, nurses, ML scientists, and hospital and clinical executive administrators when designing their clinical pathways could use this research when designing their treatment plans [47]. Furthermore, academics in the field of medicine, nursing, paramedics, hospital executive administration, patient access, and information technology will benefit from this systematic review as it allows them to build on the existing relevant literature.

## Discussion

### Principal Findings

The evolution of AI in nursing has transitioned from early symbolic AI, using rule-based algorithms and fuzzy logic, to modern data-driven approaches such as ML and ANNs and is now exploring hypothetical future waves such as artificial general intelligence. AI applications in nursing include the Rothman Index for patient acuity and risk assessment, social robots such as Sophia and LOVOT for older adults’ companionship, telerobots for remote patient interaction, and NLP for enhancing decision-making and patient communication. RPA and ML are used to automate repetitive tasks and improve diagnostic accuracy, while AI-powered tools such as chatbot assistants and wearable monitoring systems assist in patient care and safety. Case studies demonstrate AI’s role in supporting, rather than replacing, nursing functions, enhancing efficiency, and allowing nurses to focus more on direct patient care. The success or failure of the medical AI solution will depend on how closely system architects collaborate with real-world nurses in health care fields, as they are needed to work closely together to assess and evaluate which technologies will be prioritized for development [17].

Inadequate evaluation, careless supervision, a lack of fundamental nursing knowledge, a lack of service awareness, and unlawful activity by nursing personnel are all contributing factors to poor nursing care. Inadequate evaluation forces nursing staff members to advance their own skills, be able to analyze certain nursing conditions, and act quickly to take timely, scientifically sound action. Poor nursing is also largely caused by a lack of thorough inspection by nursing staff. Individuals who make nursing errors because of inadequate nursing knowledge should enhance their own skills and training. Ultimately, the nursing profession is a service sector. The essential spirit of service is required when treating patients. It is imperative that corresponding services are rendered completely in compliance with industry standards, and any illicit activities are forbidden. To address this, the use of AI alone will not mitigate those issues; instead, the health care facility’s relevant departments must develop a mechanism to penalize slack investigation and prevent the recurrence of such unfavorable circumstances [48].

### Methodological Approach Limitations

The limitations of our systematic literature review methodological approach include potential publication bias; additionally, the quality and relevance of included studies can vary, impacting the overall reliability of the findings. The search strategy may also be limited by the databases and sources selected, potentially missing relevant literature elsewhere. Furthermore, the exclusion of non–English language studies might introduce language bias. Finally, the subjective nature of data extraction and thematic analysis can lead to inconsistencies and affect the validity of the conclusions.

### Future Directions and Recommendations

Due to research that is still in the early stages of development and the considerable variation in AI types and situations, AI used in health care and nursing care is still a developing practice with minimal evidence. AI in nursing becomes a crucial component of health care delivery in general and nursing practice in particular. There is still a great deal of room for advancement with these systems in terms of ensuring not only the professional autonomy of nurses but also better access to sources of health information to maximize their use in multitasking, to cover the greatest number of factors that may affect the patient, environment, clinical practice, and various medical services. Additional research is required to determine how previous research findings using AI-based systems with virtual reality or simulated scenarios can be applied to real-world clinical nursing practice or to examine how these AI-based support systems may enhance patient safety and help nurses in specific clinical settings [28]. A blueprint of nurse involvement in the deployment of AI-based systems and applications can act as a guiding reference and is an area that is worth further research and exploration.

AI has become a game-changing technology that is transforming several industries, the health care industry most notably. It is essential for diagnosing uncommon genetic diseases, streamlining patient care in mental health clinics, supporting clinical judgment, and revolutionizing pathological research. However, the growing use of AI in health care also raises difficult moral, practical, and legal questions, especially in light of the General Data Protection Regulation framework in Europe. The significance of understanding data owner rights and developing moral guidelines for AI use in medical applications, particularly nursing, is another area for future research. Comprehending the ethical discussion around AI helps health care and nursing professionals create moral AI procedures for practice and assists in navigating the complex landscape of AI-driven health care regulations, ethical issues, and data protection [49]. AI presents several risks, particularly in the context of deep reinforcement learning–based mobile robot assistants. Ensuring safety in environments where humans and robots interact is crucial, especially when autonomous mobility robots rely on deep reinforcement learning for navigation and decision-making. This is particularly important in health care settings, where hospital patients using these AI-driven mobility assistants may face potential hazards that require careful evaluation and mitigation [50]. Therefore, there is an opportunity to further research the risks associated with the use of AI in nursing.

### Conclusions

To fully benefit from AI technology, nurses will need to develop their ability to collaborate with data scientists. Although computer science and nursing are two separate fields, knowledge and skill transfer between the two is crucial as technology develops so that nurses may learn to interpret the data. In the future, nurses will play the role of coaches who will assist people in managing their health and achieving better results. The provision of touch and building connections with patients are the foundations of the nursing profession and their role in patient care, and they will never be fully replaced by AI tools or robots, especially when collecting medical information, such as heart monitoring, urinalysis, and range-of-motion analysis [9].

As emerging AI technologies take over some of the jobs that nurses already do, nursing will be impacted. Although technology will alter the way nurses spend their time providing patient care, nurses will still be required. The nurse will acquire new ways of thinking about and processing information; they will become information integrators, health coaches, and providers of human care, assisted by AI technologies rather than being replaced by them [9]. Current research and implementations demonstrate the effectiveness and promise of AI in nursing practice. However, they do not eliminate the need for field supervision and emotional support from humans [51]. Therefore, and to answer the research question, “Will AI change the role of nurses in patient care?” based on the outcome of this literature review, the answer is “yes” with a varying extent depending on the AI tool in use by the nursing professionals.

Interest in incorporating AI into nursing practice will not go away, although its technological potential is not well understood. It is highly recommended that academic institutions and professional associations implement suitable educational and training initiatives. It is imperative that nurses enhance their comprehension of fundamental AI and its integration into nursing practice [52].

## Appendix

Multimedia Appendix 1 Systematic review analysis.

Multimedia Appendix 2 Critical assessment of the literature.

Multimedia Appendix 3 PRISMA Checklist.

## Abbreviations

AI: artificial intelligence
ANN: artificial neural network
C4W: CARES4WOUNDS
DFU: diabetic foot ulcer
EMR: electronic medical record
ML: machine learning
NLP: natural language processing
RPA: robotic process automation
SWAT: Specialized Workforce for Acute Transport

## Data Availability

All data generated or analyzed during this study are included in this published article and its supplementary information files.
